# Supplementary material for: Global hypo-methylation in a proportion of glioblastoma enriched for an astrocytic signature is associated with increased invasion and altered immune landscape
Source: eLife. 2022 Nov 22;11:e77335. doi: 10.7554/eLife.77335 (PMC9681209; doi:10.7554/eLife.77335)
Supplement: Figure 2—source data 1. [file elife-77335-fig2-data1.zip › Figure_2_source_data_1/Figure_2C/homerResults/motif9.similar.html]

motif9

## Information for motif9

T
A
G
C
T
C
A
G
A
T
G
C
T
A
G
C
T
A
C
G
T
G
A
C
A
G
T
C
A
T
G
C
A
T
G
C
A
T
G
C
A
T
G
C
G
A
C
T
C
A
T
G
T
A
C
G
A
T
C
G
T
A
C
G
C
A
G
T
A
T
C
G
A
T
G
C
T
C
A
G
G
T
A
C
T
A
C
G
T
A
G
C
C
T
G
A
A
T
G
C
A
T
G
C
T
A
G
C
T
G
A
C
A
C
T
G
T
A
C
G
A
C
T
G
A
T
C
G
T
A
C
G
T
C
A
G
C
T
G
A
A
T
C
G
T
G
A
C
C
T
A
G
A
T
G
C
T
A
G
C
A
T
G
C
G
T
C
A
A
G
T
C
A
T
G
C
A
T
G
C
A
T
G
C
G
C
A
T
T
A
C
G
A
C
T
G
T
A
C
G
  
Reverse Opposite:  

A
T
G
C
T
A
G
C
A
T
G
C
C
G
T
A
A
T
C
G
T
A
C
G
T
A
C
G
T
C
A
G
C
A
G
T
T
A
C
G
A
T
C
G
T
A
C
G
A
G
T
C
A
C
T
G
T
A
G
C
G
A
C
T
A
G
T
C
A
T
G
C
A
T
G
C
A
G
T
C
A
T
G
C
G
T
A
C
A
C
T
G
A
T
C
G
T
A
C
G
T
A
C
G
G
A
C
T
A
T
C
G
A
T
G
C
C
A
T
G
A
G
T
C
T
A
C
G
T
A
G
C
G
T
C
A
A
T
G
C
T
A
G
C
A
T
G
C
G
A
T
C
C
T
G
A
T
A
C
G
A
T
C
G
A
T
C
G
T
A
C
G
T
C
A
G
A
C
T
G
A
T
G
C
A
T
C
G
T
A
C
G
A
G
T
C
A
T
C
G
  

|  |  |
| --- | --- |
| p-value: | 1e-17 |
| log p-value: | -4.123e+01 |
| Information Content per bp: | 1.480 |
| Number of Target Sequences with motif | 13.0 |
| Percentage of Target Sequences with motif | 1.11% |
| Number of Background Sequences with motif | 1.1 |
| Percentage of Background Sequences with motif | 0.02% |
| Average Position of motif in Targets | 94.2 +/- 43.1bp |
| Average Position of motif in Background | 34.0 +/- 0.0bp |
| Strand Bias (log2 ratio + to - strand density) | -0.6 |
| Multiplicity (# of sites on avg that occur together) | 1.23 |
| Motif File: | file (matrix) reverse opposite |

### Similar de novo motifs found

|  |  |  |  |  |  |  |  |
| --- | --- | --- | --- | --- | --- | --- | --- |
| Rank | Match Score | Redundant Motif | P-value | log P-value | % of Targets | % of Background | Motif file |
| 1 | 0.684 | T G A C C T A G A T G C T A G C A G T C A G T C A G T C T C G A A T G C A G T C A C G T A G T C T A C G T A C G A T C G A T C G A C G T A T C G T A G C T C A G A T G C A T G C T A G C A G C T A G T C G A T C A T C G A T G C G A C T A G T C A T C G A T C G A T C G T C A G G A C T T A C G T G A C T A C G A G T C T A C G | 1e-12 | -29.505524 | 0.85% | 0.00% | motif file (matrix) |
| 2 | 0.636 | A G T C T A C G A G T C A T G C T A G C G A C T A C G T T A G C A T G C A T G C G C T A A T C G T A C G A T C G A T C G T A C G C G A T A T C G G T A C C A T G A G T C A T G C A T G C C G A T A G T C A T G C A G T C A G T C G C T A A G C T | 1e-9 | -22.157850 | 0.68% | 0.02% | motif file (matrix) |
